# LncRNA levels in the central nervous system as novel potential players and biomarkers in amyotrophic lateral sclerosis

**DOI:** 10.1016/j.ncrna.2025.05.017

**Published:** 2025-06-09

**Authors:** Tresa López-Royo, Laura Moreno-Martínez, Gabriel Rada, Sofía Macías-Redondo, Ana Cristina Calvo, Alberto García-Redondo, Raquel Manzano, Rosario Osta

**Affiliations:** a Department of Anatomy, Embryology and Animal Genetics, University of Zaragoza, Centro de Investigación Biomédica en Red de Enfermedades Neurodegenerativas (CIBERNED), Agroalimentary Institute of Aragon (IA2), Institute of Health Research of Aragon (IIS), Calle Miguel Servet 177, 50013, Zaragoza, Spain; b Instituto Aragonés de Ciencias de la Salud (IACS), Centro de Investigación Biomédica de Aragón (CIBA), 50009, Zaragoza, Spain; c Neurology Department, ALS Unit, Hospital 12 de Octubre Health Research Institute (i+12), CIBERER U-723 (Instituto de Salud Carlos III) Avda, Córdoba, s/n , Madrid, 28041, Spain

**Keywords:** ALS, Neat1, H19, Malat1, Gas5, Myoparr

## Abstract

Research in amyotrophic lateral sclerosis (ALS) faces major burdens, including the urgent need for sensitive and specific biomarkers, the identification of novel and effective therapeutic targets and a deeper understanding of the mechanisms driving the disease. In this line, long non-coding RNAs (lncRNAs) have emerged as promising candidates due to their regulatory role in a variety of important biological processes such as RNA metabolism, neuroinflammation, apoptosis or proteostasis.

This study aims to elucidate the expression profile of 14 lncRNAs in both the SOD1^G93A^ mouse model and ALS patients. Different stages of the disease (presymptomatic, symptomatic and terminal) and 3 regions of the central nervous system (CNS) differentially affected by ALS (spinal cord, brainstem and frontal cortex) were included in the experimental design.

In SOD1^G93A^ mice, all 14 lncRNAs exhibited differential expression patterns influenced by sex, age, and region, except for Malat1, Neat1, and H19, which displayed consistent expression patterns (Malat1 was decreased, while Neat1 and H19 were increased). These patterns were most prominent in the spinal cord, where lncRNAs were overall down-regulated. In contrast, in the brainstem and frontal cortex, lncRNAs were predominantly up-regulated. Notably, *Gas5* expression levels in frontal cortex and spinal cord at the terminal stage correlated with the onset and progression of motor coordination and strength decline. Additionally, three lncRNAs (*Gas5*, *Neat1* and *Myoparr*) were found to significantly correlate with survival.

In human ALS samples, increased levels of *NEAT1* and *SNHG16* were observed in the brainstem, and of *MEG3* and *H19* in the frontal cortex, whereas *MALAT1* levels were decreased in frontal cortex.

In conclusion, this work supports lncRNAs as promising candidates as novel players and potential biomarkers in ALS and highlights SOD1^G93A^ mice as a good model to study lncRNAs in the CNS in the context of this disease.

## 1 Introduction

Amyotrophic Lateral Sclerosis (ALS) is a neurodegenerative disease (NDD) characterized by the progressive and selective loss of motor neurons, accompanied by the atrophy and paralysis of voluntary muscles. In spite of being considered a rare disease, ALS is one of the most common NDDs [1]. Unfortunately, there is no cure for ALS, and patients face a limited life expectancy after the onset of the first symptoms, which may go unnoticed in the early stages of the disease. In addition, the clinical heterogeneity and the lack of specific biomarkers hamper both diagnosis and clinical management, and highlight the need for a better understanding of the disease to facilitate development new biomarkers and effective therapeutic strategies [2].

Despite considerable efforts, the field is still far from fully understanding ALS pathogenesis. Indeed, most ALS cases are sporadic (sALS), while only 5–10 % are caused by inherited genetic mutations and can be considered as familial forms (fALS). Among the inherited forms, mutations in the *SOD1* (15–20 %)*, C9ORF72* (30–40 %), *FUS* (5 %) and *TARDBP* (4 %) genes are the most common. These genes can also be found mutated in sporadic forms, although in a smaller percentage, highlighting the fundamental role of RNA metabolism in the pathophysiology of the disease [3].

In recent years, both small (<200 bps) and long (>200 bps) non-coding RNAs have emerged as molecules with a strong involvement in RNA metabolism and ALS [4,5]. In this context, lncRNAs raise a great interest to be investigated in ALS due to their significant role and abundance in the central nervous system (CNS), their misregulation in biological samples of patients and their ability to regulate gene expression at pre-, post- and transcriptional levels [6].

In this line, *Neat1* was the first lncRNA linked to ALS, being upregulated in the spinal motor neurons of six sporadic ALS patients [7]. Interestingly, lncRNAs are more abundant in the CNS than in any other body system, participating in its evolution, adaptability and maintenance, as well as in the differentiation and operation of different neuronal subtypes.

In 2018, RNA-seq of peripheral blood mononuclear cells from sALS patients showed three times more differentially expressed lncRNAs than mRNAs. These data were confirmed in spinal cord samples from a different patient cohort [8]. Since then, a few other studies have attempted to address the study of the role of lncRNAs in ALS, although it remains essentially a nascent field of research [[9], [10], [11], [12], [13], [14]].

In this work, we explored the expression of 14 lncRNAs in the murine model of ALS SOD1^G93A^. These lncRNAs were selected from the existing literature due to their differential expression in other neurodegenerative or neuromuscular diseases and processes. Specifically, we analysed the expression of the lncRNAs Meg3, Hotair, Malat1, Gas5, Neat1, Myhas, Xist, Myoparr, CDR1os, Snhg1, Snhg16, Miat, Pvt1 and H19 in brain and spinal cord areas from SOD1^G93A^ mice in comparison with B6.SJL control mice, at presymptomatic (60 days of age), symptomatic (90–100 days of age) and late symptomatic (120 days of age) stages of the disease. Our results evidenced a distinct lncRNA expression pattern in the CNS of ALS mice. Furthermore, lncRNA expression in the terminal stage correlated with the performance in functional tests (particularly for *Gas5*) as well as survival (for *Gas5*, *Neat1* and *Myoparr*). Finally, the differential expression pattern of five of these lncRNAs (*NEAT1*, *SNHG16*, *MEG3*, *H19* and *MALAT1*) was confirmed in the frontal cortex and brainstem of sporadic ALS patients. Altogether this work deepens our understanding of lncRNAs as potential disease-modifying agents, biomarkers or side effectors of the ALS neurodegenerative process.

## 2 Materials and methods

### 2.1 Animals

Wildtype (WT) mice on a B6SJL genetic background and transgenic SOD1^G93A^ ALS mouse model on a mixed B6SJL background were used for all the experimental procedures. WT and transgenic SOD1^G93A^ mice were obtained by crossbreeding hemizygous B6.SJL-Tg SOD1^G93A^ males from The Jackson Laboratory (Bar Harbor, ME, USA) with B6.SJL females from Janvier Labs (Saint-Berthevin Cedex, France). The offspring genotype was determined by PCR amplification of DNA extracted from tail tissue as described in The Jackson Laboratory protocol.

Mice were hosted at the animal facilities in Centro de Investigación Biomédica de Aragón in a pathogen-free environment and under a standard light/dark (12:12) cycle. Food and water were administered *ad libitum*. All experimental procedures were approved by the Ethic Committee for Animal Experiments from the University of Zaragoza (PI29/13 and PI08/19) and compiled according to the institutional and international guidelines for the use of laboratory animals.

A total of 153 mice were included in this study. 130 animals were used for the characterization of the distinctive lncRNA expression pattern of SOD1^G93A^ mice: with 64 animals in the study group (n = 34 for spinal cord and n = 30 for brainstem and frontal cortex; sex balanced littermates); and 66 animals in the control group (n = 34 for spinal cord and n = 30 for brainstem and frontal cortex; sex balanced littermates). Finally, 23 SOD1^G93A^ mice (n = 11 male, n = 12 female) were used for the study correlations with functional tests and life expectancy.

### 2.2 Functional tests

Motor coordination, strength and balance were assessed using a rotarod (ROTA-ROD/RS, LE8200, LSILETICA Scientific Instruments; Panlab, Barcelona, Spain) [[15], [16], [17]]. Animals were placed onto the cylinder at a constant speed of 14 rpm once a week from week 8 until humane endpoint.

Muscle strength and endurance were evaluated by the hanging-wire test [[15], [16], [17]]. Mice were placed on a wire lid of a conventional housing cage and turned upside down. The latency from the beginning of the test until the mouse could no longer hold was measured for each animal.

In both tests, each animal had three attempts to stand for a maximum of 180 s per trial, and the longest latency was recorded (Supplementary Tables 1 and 2). The timepoint (expressed in weeks) at which each animal was unable to complete the full test was considered to be the onset of symptoms or functional decline. Disease duration was also determined by calculating the number of weeks from the onset of symptoms to the humane endpoint.

### 2.3 Survival study

Survival time of animals was registered as they reached the humane endpoint (HEP) (Supplementary Table 3). HEP for this ALS mouse model was defined as the failure to right after laying the mouse on its side for 30 s as defined in Ludolph A et *al* (2010) [18].

### 2.4 Mice sample collection and RNA extraction

WT and transgenic SOD1^G93A^ mice were euthanized by CO_2_ inhalation at different disease stages: presymptomatic (at 60 days of age), symptomatic (at 90–100 days of age), late symptomatic (120 days of age) and humane endpoint stages. Spinal cord and brain tissues were rapidly removed and stored at −80 °C until processed. Detailed information on the animals used in each experiment, stage, and group is provided in Supplementary Table 4.

For RNA extraction, spinal cord and brain samples (previous isolation of frontal cortex and brainstem areas) were homogenized in Trizol reagent using Tissue Lyser LT (Qiagen; Hilden, Germany). Total RNA was isolated using Direct-zol™ RNA MiniPrep Kit (Zymo Research; Irvine, CA, USA), according to the manufacturer's protocol.

### 2.5 Human sample collection and RNA extraction

Brain tissue samples from patients were collected, processed and provided by the CIEN Tissue Bank, CIEN Foundation, *Instituto de Salud Carlos III* and *Biobanco en Red de la Región de Murcia* (BIOBANC-MUR). All subjects provided written informed consent and *El Comité de Ética de la Investigación de la Comunidad de Aragón* (CEICA) (Ref. PI17/0025, modified on June 2023) and *El Comité Científico del banco de tejidos de la Fundación CIEN* (Ref. CCS17003, modified CEI PI 79_2023) approved this research. For detailed clinical characteristics, please refer to Supplementary Table 5.

RNA extraction from human samples was carried out as previously described in Oros et al., 2017 [19]. The quality and concentration of each sample were measured with Nanodrop ND-1000 spectrophotometer (Thermo Fisher Scientific).

### 2.6 Real-Time PCR (RT-qPCR)

RNA was retrotranscribed with the High Capacity cDNA Reverse Transcription Kit (Thermo Fisher Scientific; Waltham, MA, USA). Quantitative PCR was performed from diluted cDNA in triplicates using the Quant Studio™ 3 Real Time PCR Instrument (Thermo Fisher Scientific). Primers were self-designed using the NCBI primer designing tool (Thermo Fisher Scientific), see Supplementary Table 6.

Relative gene expression was calculated by the 2^-ΔΔCT^ method, using *GAPDH* and *ACTB* as housekeeping genes [20].

### 2.7 Statistical analysis

GraphPad Prism version 8.0.1 software was used for the statistical analysis and illustration of figures. Results are shown as the mean value ± the standard error of the mean (SEM) or standard error (SD) as indicated. In expression studies of murine and human samples, outliers for each lncRNA and group were identified as those outside the expected normal limits calculated according to Ref. [21]:

Upper limit = (75th percentile) + (1.5∗(75th percentile – 25th percentile))

Lower limit = (25th percentile) − (1.5∗(75th percentile – 25th percentile))

Student's t-test was used to stablish significant differences between ALS and age-matched control groups in this study. In animal survival studies, outliers were identified by Grubbs' test. Pearson or Spearman correlation tests (depending on the normal or non-parametric distribution of data) were performed to asses correlation of survival or functional tests with lncRNA levels. Differences were considered statistically significant if p < 0.05 (∗), p < 0.01 (∗∗) or p < 0.001 (∗∗∗).

## 3 Results and discussion

### 3.1 Selection of lncRNAs for the study

**Fig. 1:**
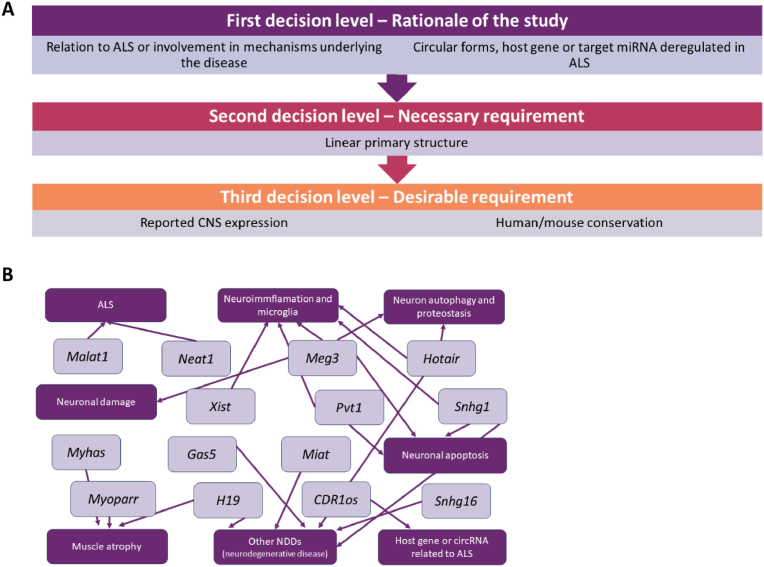
**Selection of lncRNAs potentially involved in ALS. (A)** Selection criteria **(B)** LncRNAs selected from the study and associated molecular mechanisms.

To identify potential lncRNAs involved in ALS pathophysiology, an extensive literature review was conducted. Selection criteria for this search included: 1) linear structure, 2) relation to ALS or involvement in mechanisms underlying the disease (neurodegeneration, neuroinflammation, muscle atrophy, proteostasis, etc), 3) circular forms or host gene differentially expressed in ALS, 4) conservation between human and mouse species, and 5) lncRNA expression in the CNS (Fig. 1a). From this study, 14 lncRNAs were selected (Table 1, Fig. 1b).

**Table 1:** LncRNAs selected for this study.

LncRNA	NCBI gene IDs∗	Reason for study
*Meg3*	17263 (Mm, mouse)	Involved in microglial activation and neuroimmflamation [22]. Also involved in neuron autophagy, apoptosis, motor neuron differentiation and neuronal damage [[23], [24], [25], [26], [27]].
	55384 (Hs, human)	
*Hotair*	100503872 (Mm)	Linked to neurodegeneration, proteostasis, activation of microglia and Parkinson's disease [[28], [29], [30]].
	100124700 (Hs)	
*Malat1*	72289 (Mm)	Associated with ALS [14,31,32].
	378938 (Hs)	
*Gas5*	14455 (Mm)	Related to neurodegenerative diseases such as AD, PD, MS and myasthenia gravis [[33], [34], [35], [36], [37], [38], [39]].
	60674 (Hs)	
*Neat1*	66961 (Mm)	Involved in ALS pathophysiology [7,[40], [41], [42], [43]].
	283131 (Hs)	
*Myhas*	102633540 (Mm)	Associated with muscle atrophy and myogenesis [44].
	100128560 (Hs)	
*Xist*	213742 (Mm)	Linked to other neuropathies with common hallmarks such as neuroinflammation.
	7503 (Hs)	
*Myoparr*	114004354 (Mm)	Related to muscle atrophy [45,46].
	114004358 (Hs)	
*CDR1os*	331424 (Mm)	*CDR1* gene presents copy number variations in ALS [47]. Circular form (*ciRS7*) is decreased in SOD1^G93A^ CNS [48] and involved in brain development, neuroinflammation and myogenesis [[49], [50], [51]].
	∗∗only circular form (Hs)	
*Snhg1*	83673 (Mm)	Related to apoptosis in neurons, microglia and other NDDs such as PD [[52], [53], [54], [55], [56], [57]].
	23642 (Hs)	
*Snhg16*	66293 (Mm)	Potential biomarker in myasthenia gravis [58]. Related to IL-10 (implicated in ALS [59]) in this disease.
	100507246 (Hs)	
*Miat*	330166 (Mm)	Involved in AD and PD [[60], [61], [62]]. Regulates *VEGFA* [63] (VEGF is a biomarker for ALS used in clinical trials [64]).
	440823 (Hs)	
*Pvt1*	19296 (Mm)	Associated with neuroinflammation and neuronal cell apoptosis [65,66].
	5820 (Hs)	
*H19*	14955 (Mm)	Regulator of myogenesis and muscle atrophy and implicated in other NDDs [35,[67], [68], [69], [70], [71], [72]].
	283120 (Hs)	

AD: Alzheimer's disease; PD: Parkinson's disease; MS: multiple sclerosis.

### 3.2 Distinct lncRNA expression pattern in the central nervous system of SOD1^G93A^ ALS mice

**Fig. 2:**
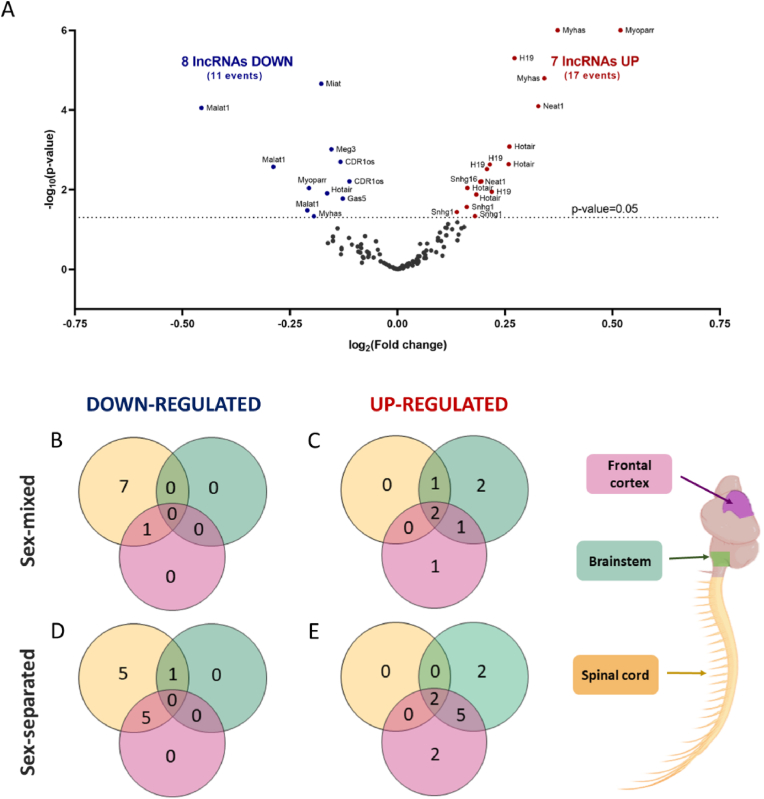
**Differentially expressed lncRNAs in the central nervous system of SOD1^G93A^ ALS mice. (A)** Volcano plot of differentially expressed genes identified between SOD1^G93A^ mice and age-matched control group. Each point represents the fold-change of ALS mouse model at a given stage (pre-symptomatic, symptomatic or late symptomatic) and tissue (spinal cord, brainstem and frontal cortex). The blue dots denote down-regulated gene expression, the red dots denote up-regulated gene expression. **(B**–**E)** Venn diagrams of down- and up-regulated lncRNAs taking into account sex-mixed groups of ALS and WT mice (B down-regulated, C up-regulated) and separating by sex (D down-regulated, E up-regulated). Yellow, green and purple colours correspond to the spinal cord, brainstem and frontal cortex, respectively. N = 24 (spinal cord) and N = 20 (brainstem and frontal cortex) per stage, balanced in sex and genotype. Results shown were obtained by RT-qPCR; supplemental information for this figure can be found in Supplementary Fig. 1 and Supplementary Table 7.

**Fig. 3:**
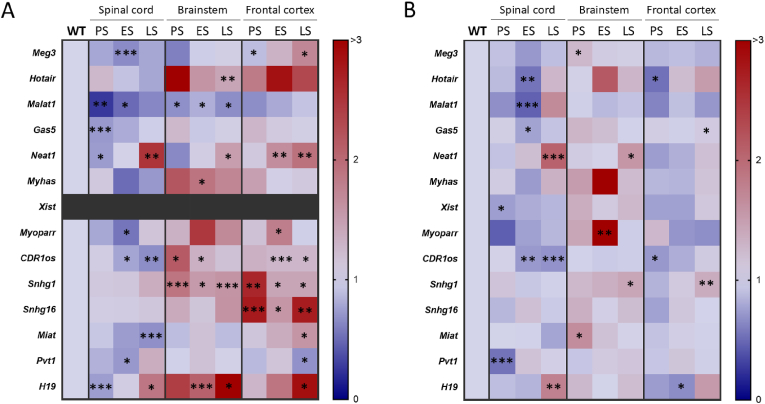
**LncRNA expression pattern in the central nervous system of SOD1^G93A^ ALS mice.** Heat map of lncRNA fold change of male **(A)** and female **(B)** SOD1^G93A^ mice in different regions of the central nervous system. LncRNA fold change is expressed as a value rated from 0 to >3, calculated as the ratio of RNA expression levels by RT-qPCR in ALS mice compared to their age-matched WT littermates. Xist expression is absent in males, as its transcription is restricted to individuals with two X chromosomes. N = 24 (spinal cord) and N = 20 (brainstem and frontal cortex) per stage, balanced in sex and genotype. Supplemental information for this figure can be found in Supplementary Table 7. PS: presymptomatic stage (P60), ES: symptomatic stage (P90-P100), LS: late symptomatic stage (P120). ∗p < 0.05, ∗∗p < 0.01, ∗∗∗p < 0.001.

The selected lncRNAs were analysed by RT-qPCR in the spinal cord, brainstem and frontal cortex of SOD1^G93A^ and WT mice at the presymptomatic (P60), symptomatic (P90-100) and late symptomatic stages (P120). Results evidenced a distinct expression pattern of SOD1^G93A^ mice, with differences in all of the 14 studied lncRNAs (Fig. 2A and Supplementary Fig. 1), pointing to a substantial dysregulation of these molecules in the nervous system in this condition. These differences were age-, sex-, region-dependent and lncRNA-specific (Fig. 2, Fig. 3). These data contrast with the pattern observed for other non-coding RNAs such as circular RNAs, which show a general decrease in expression within the CNS [48,73].

Notably, SOD1^G93A^ males exhibit accelerated disease progression compared to females, which was reflected in different lncRNA expression patterns in both sexes, with greater expression changes in males. Indeed, the number of differentially expressed lncRNAs was higher when analysing both sexes separately (Fig. 2D and E) (100 % of lncRNAs, 53 events) as compared to together (Fig. 2A–C) (85.71 %, 28 events).

Moreover, alterations in lncRNA expression were observed in all 3 regions with the highest number of changes in the spinal cord, consistent with the predominant affection of motor neurons in this region in SOD1^G93A^ mice (Fig. 3). Affection of brainstem neurons has also been described in this model, albeit to a lesser extent, while involvement of the frontal cortex has scarcely been reported. Surprisingly, SOD1^G93A^ mice also showed significant changes in lncRNA expression in the frontal cortex, although it remains unclear whether these changes represent a protective response or a pathological event that remains subclinical. Interestingly, male and female expression profiles were more similar in the regions with higher disease involvement (spinal cord > brainstem > frontal cortex).

Overall, lncRNAs were found to be downregulated in the spinal cord, while upregulated in the frontal cortex of males and the brainstem (Fig. 2, Fig. 3). Altogether these results highlight the anatomical differences in lncRNA expression, which may reflect/mirror the varying vulnerability of the diverse CNS regions in this disease, related or not to the involvement of distinct molecular pathways or lncRNA functions in each area. As exceptions, *Malat1*, *Neat1* and *H19*, which were differentially expressed (*Malat1* decreased, *Neat1* and *H19* increased) in SOD1^G93A^ CNS in a region-independent manner (Fig. 3). These lncRNAs, which maintain a consistent expression pattern across all analysed regions, might be associated with broader pathophysiological events characteristic of this condition.

### 3.3 Gas5 levels in different regions correlate with functional decline onset, disease duration and survival in SOD1^G93A^ mice

In the search for potential monitoring or prognostic biomarkers among the differentially expressed lncRNAs, cortex, brainstem and spinal cord samples were collected from 23 SOD1^G93A^ mice at the terminal stage (humane endpoint). LncRNA expression levels were then correlated with key preclinal parameters, including lifespan, symptom onset (marked by functional decline) and disease duration. In terms of functional decline, and consistently with previous findings [74], muscle strength impairment (as assessed by failure in the hanging wire test) occurred earlier than deficits in motor coordination and balance, which were evaluated using the rotarod test (Supplementary Tables 1 and 2).

This analysis revealed that *Meg3* and *Miat levels* in spinal cord and *Gas5* expression in frontal cortex correlated to motor coordination and balance performance in male SOD1^G93A^ mice. Similarly, *Gas5* and *Snhg1* levels in the spinal cord and brainstem, respectively, correlated to female muscle strength weakening. In addition, *Hotair*, *Myhas (spinal cord)*, *Gas5 (brainstem)*, *Neat1* and *Myoparr (frontal cortex)* levels correlated with survival (Table 2).

**Table 2:** **Correlations determined for lncRNAs.** Correlation plots for lncRNAs other than *Gas5* and *Myoparr* are shown in Supplementary Fig. 2c–l.

Measured parameters	LncRNA	Sample	Pearson r (P)/Spearman r (S)	p-value
Motor coordination and balance failure onset (symptom onset by rotarod)	*Meg3*	Male spinal cord	0.6692 (P)	0.0243∗
	*Miat*	Male spinal cord	0.6682 (P)	0.0246∗
	*Gas5*	Male frontal cortex	−0.7080 (P)	0.0148∗
Disease duration according to motor coordination and balance failure (disease duration by rotarod)	*Meg3*	Male spinal cord	−0.7467 (P)	0.0083∗∗
	*Miat*	Male spinal cord	−0.7180 (P)	0.0128∗
	*Gas5*	Male frontal cortex	0.6936 (P)	0.0179∗
Muscle strength failure onset (symptom onset by hanging-wire)	*Gas5*	Female spinal cord	0.6292 (S)	0.0318∗
	*Snhg1*	Female brainstem	0.7259 (P)	0.0075∗∗
Disease duration muscle strength failure (disease onset by hanging-wire)	*Gas5*	Female spinal cord	−0.6456 (S)	0.0268∗
	*Snhg1*	Female brainstem	−0.8095 (P)	0.0014∗∗
Survival	*Hotair*	Male and female spinal cord	0.4668 (P)	0.0247∗
	*Myhas*	Male and female spinal cord	0.4825 (P)	0.0197∗
	*Gas5*	Male and female brainstem	−0.5840 (P)	0.0034∗∗
	*Neat1*	Male frontal cortex	−0.6769 (P)	0.0221∗
	*Myoparr*	Male frontal cortex	0.8270 (P)	0.0060∗∗

**Fig. 4:**
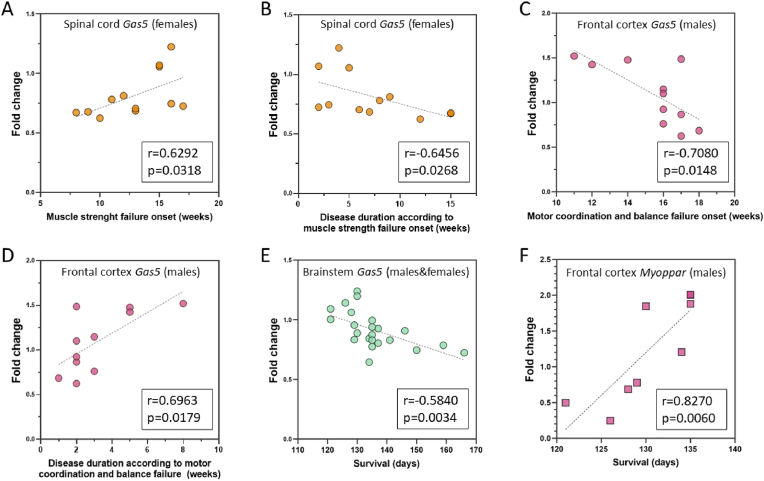
**LncRNAs *Gas5* and *Myoparr* as potential modifiers of ALS progression in SOD1^G93A^ mice. (A,B)***Gas5* levels in the spinal cord of females at the humane endpoint correlate with muscle strength failure onset (A) and disease duration (B). **(C,D)***Gas5* levels in the frontal cortex of terminal SOD1^G93A^ males correlates with motor coordination and balance failure onset (C) and disease duration (D). **(E)***Gas5* levels in the brainstem at the humane endpoint correlate with SOD1^G93A^ mice survival. **(F)***Myoparr* levels in the frontal cortex of terminal SOD1^G93A^ males correlates with survival. For this experiment, N = 23 SOD1^G93A^ mice (n = 11 males, n = 12 females) at the humane endpoint. Results were obtained by RT-qPCR.

Remarkably, *Gas5* levels correlated with the outcomes of both behavioral tests as well as survival (Fig. 4). Specifically, elevated *Gas5* expression in the spinal cord of females were concomitant with a delayed onset and a faster progression of strength symptoms (Fig. 4A and B). Conversely, in males, higher levels of *Gas5* in the frontal cortex correlated with earlier onset and slower progression of motor and coordination symptoms (Fig. 4C and D). Furthermore, for both sexes, animals displaying higher levels of *Gas5* in the brainstem exhibited shorter survival (Fig. 4E). These findings are in line with ours and other groups’ previous findings of gender- and CNS anatomic-specific expression of lncRNAs likely associated to distinct roles in each cell type, gender and/or tissue both in physiological and pathological conditions (unpublished own data and [[75], [76], [77], [78], [79], [80], [81]]). In this sense, lncRNA *Gas5* has been implicated in the regulation of several metabolic processes relevant to ALS CNS pathology such as neuroinflammation, mitochondrial damage and neuronal death by apoptosis [35,38,39,[82], [83], [84]].

Likewise, *Myoparr* levels in the frontal cortex of terminal SOD1^G93A^ male mice have been robustly correlated with survival (Fig. 4F). As far as we are aware, this lncRNA has only been described in the context of skeletal muscle [45,46,[85], [86], [87], [88]]. These results not only demonstrate that it is expressed in the CNS, but it could also reflect ALS pathological traits in this tissue. In this sense, *Myoparr* has been shown to negatively regulate *GDF5* gene expression in denervated skeletal muscle in mice [46], so that knocking down *Myoparr* leads to increased levels of *GDF5*. This growth factor also plays an important role in the central nervous system, with neurotrophic effects on dopaminergic neurons [89]. Precisely, loss of dopaminergic neurons in sporadic ALS patients has been linked to more rapid disease progression and associated dementia [90]. This *Myoparr*-GDF5-dopaminergic neurons axis would be in line with our findings as higher expression of *Myoparr* in the brainstem of SOD1^G93A^ mice is associated with shorter lifespan.

Interestingly, in males, correlations between lncRNAs and symptom onset and progression were detected using the rotarod test, while for females, it occurred with the hanging-wire test (Table 2). This might strengthen the differences in lncRNA expression between sexes and the variability of lncRNA function according to context and tissue type. The phenotypic differences observed in this animal model between males and females and the distinct parameters measured by each functional test should also be considered to proper outcome interpretation. The rotarod test assesses motor coordination and balance, focusing mainly on hindlimb function, whereas hanging-wire test evaluates muscle strength and endurance, with an emphasis on forelimb performance [[15], [16], [17]]. Thus, *Gas5* levels in frontal cortex might be related to motor coordination decline rate in hindlimbs, while in spinal cord it could rather be a reflection of muscle strength in the forelimbs, being these processes more important/susceptible in male and female mice respectively.

Furthermore, symptom onset was negatively correlated with disease duration, defined as the interval of time elapsed from symptom onset to the humane endpoint. Thus, mice with earlier symptom onset showed slower disease progression (Supplementary Fig. 2A and B). This finding mirrors clinical observations in ALS patients, where age of onset is a risk factor for poor prognosis. Older age of onset is associated with more aggressive forms of the disease, whereas earlier onset forms of ALS typically have slower symptom progression and a better prognosis [[91], [92], [93]].

### 3.4 NEAT1, SNHG16, MEG3, MALAT1 and H19 lncRNAs are differentially expressed in the brainstem and frontal cortex of sporadic ALS patients

To assess whether the expression patterns of lncRNAs found in SOD1^G93A^ mice were also present in ALS patients, we next analysed the expression of the homologous human lncRNAs in RNA samples derived from *post-mortem* brainstem and cortex tissues of both sALS patients and healthy controls. Homologous human lncRNAs were identified for 13 of the 14 mouse lncRNAs analysed except for *CDR1os* (Table 1).

**Fig. 5:**
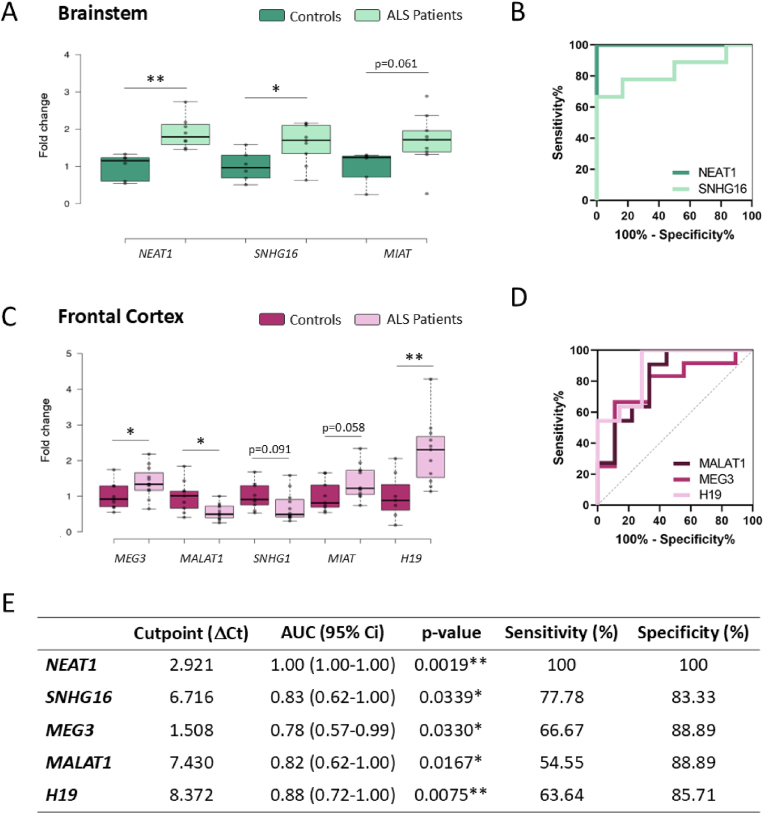
**Differential lncRNA expression in the brainstem and frontal cortex of sALS patients. (A,C)** Statistically significant lncRNA fold changes in the brainstem (A) and frontal cortex (C) of sALS patients vs. controls. **(B)** ROC curve for *NEAT1* and *SNHG16* ΔCt in brainstem. **(D)** ROC curve for *MEG3*, *MALAT1* and *H19* ΔCt in frontal cortex. **(E)** Descriptive statistics for ROC curves presented in (B) and (D). Data were obtained by RT-qPCR and are represented as mean ± SD. ROC curve plots discriminate between sALS patients and controls. ∗p < 0.05, ∗∗p < 0.01.

Of the 13 lncRNAs evaluated, five (*NEAT1*, *SNHG16*, *MEG3*, *MALAT1* and *H19*) were found to be differentially expressed in brain samples from sALS patients as compared to healthy controls, and two others (*MIAT* and *SNHG1*) showed trends towards differential expression but did not reach statistical significance (Fig. 5). Importantly, 3 out of 5 lncRNAs differentially expressed (*NEAT1*, *MEG3* and *H19*) in human samples replicated the previous findings obtained in 120-days-old SOD1^G93A^ male mice. 120-days-old males in this model are close to the humane endpoint and would therefore be the most comparable to *post-mortem* samples. Moreover, *Malat1* levels were not reduced in the cortex of ALS mice but were decreased in the spinal cord and brainstem.

These results corroborate previous research indicating high functional conservation of lncRNAs between mammals [[94], [95], [96]]. It also underlines the potential value of this animal model to explore both ALS pathological mechanisms and therapies for the disease. This is even more relevant considering that the patients were sporadic and not familial ALS patients with mutations in the *SOD1* gene.

In particular, RNA levels of the lncRNAs *NEAT1* and *SNHG16* were found to be increased in ALS brainstem (Fig. 5A), while *MEG3* and *H19* were upregulated and *MALAT1* downregulated in frontal cortex (Fig. 5C). Interestingly, these 5 lncRNAs have been associated with the MAPK signalling pathway, which has been shown altered in ALS and related to some of the main pathological mechanisms operating in this disease such as neuroinflammation or neuronal apoptosis [[97], [98], [99], [100], [101], [102], [103]].

Moreover, ROC curve analysis was performed to evaluate the potential power of these lncRNAs to identify brains from ALS patients (Fig. 5B–D,E). Importantly, *NEAT1* expression levels in the brainstem were able to discriminate ALS patients from healthy controls with 100 % specificity and sensitivity (Fig. 5B–E). Although this finding does not have direct clinical application, as a brainstem biopsy is not feasible in human patients, it remains of great interest. The consistent upregulation of *NEAT1* in all ALS brains suggests that it is a common feature of ALS cases and may represent a core mechanism of the disease that transcends variations in factors such as form, age and sex.

Among other functions, *Neat1* is a key component of paraspeckles, subnuclear bodies involved in cellular response to stress and gene regulation. Increase of *Neat1* expression, as well as paraspeckles, have been observed in neurons from other fALS models and in the spinal cord of sALS patients [41,43,[104], [105], [106]], as well as in skeletal muscle of SOD1^G93A^ mice [107]. Research continues to investigate the physiological significance of paraspeckles and *Neat1* in ALS pathogenesis, with some works suggesting a beneficial role [41] and others pointing to a potential pathogenic contribution [41,42,108,109]. In addition, at least seven paraspeckle proteins are encoded by genes mutated in familial ALS, including *TARDBP*, *FUS*, *C9ORF72*, and *MATR3* [110]. Overall, alterations in paraspeckles and *Neat1* may be a common mechanism for fALS and sALS forms. However, further research is needed, including the exploration of *Neat1* as a potential diagnostic biomarker and therapeutic target.

## 4 Conclusions

This study reveals a differential expression pattern of lncRNAs in the CNS of SOD1^G93A^ ALS mice, which was specific to age, sex, region and lncRNA. Importantly, the most affected tissues (especially the spinal cord) showed more similar patterns between sexes. Differentially expressed lncRNAs were generally downregulated in the spinal cord but upregulated in the male frontal cortex and brainstem. As exceptions, *Neat1* and *H19*, which were up-regulated across CNS regions during the late disease stages, and *Malat1*, decreased in a region-independent manner. Notably, *Neat1* levels were increased in the brainstems of sporadic ALS patients. Together with previous findings, this suggests that exploring *Neat1* as a novel diagnostic biomarker in body fluids (such as cerebrospinal fluid or blood), as well as potential therapeutic intervention target for ALS, may offer promising insights.

Furthermore, *Gas5* lncRNA expression in various CNS regions correlated with survival and functional decline onset and progression in SOD1^G93A^ murine model. This outcome, together to region influence on lncRNA expression patterns, support the hypothesis that lncRNAs operate diverse mechanisms in different tissues and CNS regions. Additionally, *Myoparr* lncRNA, previously associated only with skeletal muscle, was found to be expressed in the CNS and correlate with survival in SOD1^G93A^ male mice, suggesting that it could influence or be a reflection of CNS pathological conditions. Both *Gas5* and *Myoparr* thus emerged as candidate lncRNA modifiers of ALS disease course.

Altogether, this work underscores the relevance of lncRNAs in ALS CNS and their potential as novel biomarkers and therapeutic targets.

## CRediT authorship contribution statement

**Tresa López-Royo:** Writing – original draft, Visualization, Methodology, Investigation, Formal analysis, Data curation, Conceptualization. **Laura Moreno-Martínez:** Writing – review & editing, Methodology, Investigation, Formal analysis, Data curation. **Gabriel Rada:** Writing – review & editing, Methodology, Investigation, Data curation. **Sofía Macías-Redondo:** Writing – review & editing, Methodology, Investigation, Formal analysis, Data curation. **Ana Cristina Calvo:** Writing – review & editing, Methodology, Investigation, Formal analysis, Data curation. **Alberto García-Redondo:** Project administration, Funding acquisition. **Raquel Manzano:** Writing – review & editing, Supervision, Methodology, Investigation, Data curation, Conceptualization. **Rosario Osta:** Writing – review & editing, Supervision, Project administration, Investigation, Funding acquisition, Conceptualization.

## Data availability

The datasets produced and analysed during the present study can be obtained from the corresponding author upon reasonable request.

## Ethics approval

The corresponding certificates, referenced PI29/13, PI08/19 and P17/0025 (modified on June 2023), have been approved and obtained by the Ethics Committee for Animal Experimentation of the University of Zaragoza and the Ethics Committee for Clinical Research. Informed consent was obtained from all individual participants included in the study. In the case of the Biobanco en Red de la Región de Murcia, samples and data from patients included in this study, who gave written informed consent, were registered on the Registro Nacional de Biobancos with registration number B.0000859, and were processed following standard operating procedures with appropriate approval of the Ethical and Scientific Committees.

## Funding

This work was funded by Instituto de Salud Carlos III and 10.13039/501100008530Fondo Europeo de Desarrollo Regional (10.13039/501100002924FEDER) 'Una manera de hacer Europa' from the 10.13039/501100000780European Union, coordinated projects PI21/00372 and PI21/00286, Centro de Investigación Biomédica en Red sobre Enfermedades Neurodegenerativas (10.13039/501100015496CIBERNED, CB18/05/0037), Consolidated Groups from Gobierno de Aragón (A19_23R), The 10.13039/501100004837Spanish Ministry of Science and Innovation with funds from the 10.13039/501100000780European Union NextGenerationEU, from the Recovery, Transformation and Resilience Plan (PRTR-C17.I1) and from the Autonomous Community of Aragón within the framework of the.

Biotechnology Plan Applied to Health. T.L.-R. was supported by the 10.13039/501100023561Ministerio de Universidades from Gobierno de España (FPU19/05625).

## Declaration of competing interest

The authors hereby declare no conflicts of interest.
